# *Plasmodium falciparum* dihydroartemisinin-piperaquine failures in Cambodia are associated with mutant K13 parasites presenting high survival rates in novel piperaquine in vitro assays: retrospective and prospective investigations

**DOI:** 10.1186/s12916-015-0539-5

**Published:** 2015-12-22

**Authors:** Valentine Duru, Nimol Khim, Rithea Leang, Saorin Kim, Anais Domergue, Nimol Kloeung, Sopheakvatey Ke, Sophy Chy, Rotha Eam, Chanra Khean, Kaknika Loch, Malen Ken, Dysoley Lek, Johann Beghain, Frédéric Ariey, Philippe J. Guerin, Rekol Huy, Odile Mercereau-Puijalon, Benoit Witkowski, Didier Menard

**Affiliations:** Malaria Molecular Epidemiology Unit, Institut Pasteur du Cambodge, 5 Boulevard Monivong, BP 983, Phnom Penh, Cambodia; National Center for Parasitology, Entomology and Malaria Control, Phnom Penh, Cambodia; Department of Parasites and Insect Vectors, Institut Pasteur, Paris, France; WorldWide Antimalarial Resistance Network, Oxford, UK; Centre for Tropical Medicine and Global Health, Nuffield Department of Medicine, Oxford University, Oxford, UK

**Keywords:** Artemisinin combination therapies, Artemisinin resistance, Cambodia, Ex vivo testing, Falciparum, In-vitro testing, Malaria, Piperaquine resistance, Treatment failure

## Abstract

**Background:**

The declining efficacy of dihydroartemisinin-piperaquine against *Plasmodium falciparum* in Cambodia, along with increasing numbers of recrudescent cases, suggests resistance to both artemisinin and piperaquine. Available in vitro piperaquine susceptibility assays do not correlate with treatment outcome. A novel assay using a pharmacologically relevant piperaquine dose/time exposure was designed and its relevance explored in retrospective and prospective studies.

**Methods:**

The piperaquine survival assay (PSA) exposed parasites to 200 nM piperaquine for 48 hours and monitored survival 24 hours later. The retrospective study tested 32 culture-adapted, C580Y-K13 mutant parasites collected at enrolment from patients treated with a 3-day course of dihydroartemisinin-piperaquine and having presented or not with a recrudescence at day 42 (registered ACTRN12615000793516). The prospective study assessed ex vivo PSA survival rate alongside K13 polymorphism of isolates collected from patients enrolled in an open-label study with dihydroartemisinin-piperaquine for uncomplicated *P. falciparum* malaria in Cambodia (registered ACTRN12615000696594).

**Results:**

All parasites from recrudescent cases had in vitro or ex vivo PSA survival rates ≥10 %, a relevant cut-off value for piperaquine-resistance. Ex vivo PSA survival rates were higher for recrudescent than non-recrudescent cases (39.2 % vs. 0.17 %, *P* <1 × 10^−7^). Artemisinin-resistant K13 mutants with ex vivo PSA survival rates ≥10 % were associated with 32-fold higher risk of recrudescence (95 % CI, 4.5–224; *P* = 0.0005).

**Conclusion:**

PSA adequately captures the piperaquine resistance/recrudescence phenotype, a mainstay to identify molecular marker(s) and evaluate efficacy of alternative drugs. Combined ex vivo PSA and K13 genotyping provides a convenient monitor for both artemisinin and piperaquine resistance where dihydroartemisinin-piperaquine is used.

**Electronic supplementary material:**

The online version of this article (doi:10.1186/s12916-015-0539-5) contains supplementary material, which is available to authorized users.

## Background

Artemisinin combination therapies (ACTs), the most effective antimalarial medicines, are the mainstay of the management of uncomplicated *Plasmodium falciparum* malaria in endemic countries [1]. Over the last decade, their wide use has contributed to a reduction in the worldwide burden of malaria [1, 2]. Unfortunately, the recent emergence of *P. falciparum* resistance to artemisinin derivatives in Southeast Asia challenges malaria control and elimination efforts. Artemisinin-resistant *P. falciparum* malaria, first reported in western Cambodia in 2008–2009 [3, 4], has since been observed in Thailand, Myanmar, Vietnam, and Lao People’s Democratic Republic [5–9], as well as China [10]. Although parasites are resistant to artemisinin derivatives [11] resulting in delayed parasite clearance, ACTs remain clinically and parasitologically efficacious thanks to partner drug efficacy [9]. However, recent studies in Cambodia reported a 15–60 % rate of late clinical failures after the standard 3-day course of dihydroartemisinin-piperaquine, the recommended ACT since 2008 [12–16]. This indicates that parasites with reduced susceptibility to both artemisinin and piperaquine are now prevalent in western Cambodia and neighboring provinces.

Artemisinin resistance is currently clinically defined as a parasite clearance half-life of 5 hours or more in Southeast Asia or persistence of microscopically detectable parasites on day 3 after treatment with an ACT [9]. The corresponding in vitro phenotype is a survival rate of more than 1 % in the Ring-stage Survival Assays (in vitro RSA^0–3h^ and ex vivo RSA) [11, 17] associated with polymorphisms in the propeller domain of the *Kelch 13* gene [17–19]. In contrast, piperaquine resistance is poorly characterized. It is currently identified by late clinical failures in patients treated with standard 3-day course of dihydroartemisinin-piperaquine. However, robust evidence of parasite-dependent resistance to piperaquine is lacking as there is no reliable in vitro phenotype and no validated genetic molecular marker. Population-based analysis of in vitro susceptibility showed recent temporal increasing geometric means of inhibitory concentration 50 % (IC_50_) for piperaquine [12, 13, 20], but a demonstration of a direct association between high piperaquine IC_50_ or inhibitory concentration 90 % (IC_90_) for isolates prior to treatment and dihydroartemisinin-piperaquine failure is lacking. Moreover, IC_50_ or IC_90_ for piperaquine in day 0 isolates from recrudescent patients are distributed over a wide range, which overlaps with IC_50_ values of isolates from non-recrudescent patients. In other words, whether the temporal increase of geometric mean of IC_50_ or IC_90_ for piperaquine reflects elimination of the most susceptible parasites to piperaquine or emergence of piperaquine-resistant parasites remains unknown.

Herein, we report a novel in vitro assay – the piperaquine survival assay (PSA) – designed to mimic in vivo exposure of parasites to a pharmacologically relevant dose of piperaquine (200 nM for 48-hours as the piperaquine half-life is estimated to ~9 days) [21]. In a retrospective study, we evaluate the association between occurrence of recrudescence and in vitro PSA survival rates of C580Y K13-mutant (artemisinin-resistant), culture-adapted *P. falciparum* isolates collected at day 0 from patients treated in 2012 with dihydroartemisinin-piperaquine. In this prospective study, conducted in 2014, we explore whether the survival rate in the ex vivo PSA combined with K13 polymorphism are predictive of dihydroartemisinin-piperaquine treatment failure. Candidate molecular markers reported as associated with piperaquine-resistant *P. falciparum* are explored in parasites classified as piperaquine-resistant or piperaquine-susceptible by the in vitro PSA assay.

## Methods

### Study design and patients

#### Retrospective investigation

One hundred forty six patients with acute uncomplicated falciparum malaria were enrolled in WHO therapeutic efficacy studies conducted in 2012–2013 at health centres in western and eastern Cambodia [16]. After obtaining written informed consent from patients or parents/guardians of children, blood samples were collected before treatment into acid-citrate-dextrose tubes (Becton-Dickinson, Franklin Lakes, NJ, USA) and were then adapted to culture and in vitro susceptibility testing [11]. Patients were treated with dihydroartemisinin-piperaquine (Duo-Cotecxin®, dihydroartemisinin 40 mg and piperaquine 320 mg, Zhejiang Holley Nanhu Pharamaceutical Co. Ltd, Jiaxing, Zhejiang province, China) and followed-up for 42 days (2009 WHO protocol) [16]. The proportion of *P. falciparum* recrudescent infections at day 42, after PCR-correction, was assessed, along with blood piperaquine concentrations at day 7 [16].

Ethical approvals were obtained from the National Ethical Committee for Health Research of the Cambodian Ministry of Health. The trial was registered at the Australian New Zealand Clinical Trials Registry (ACTRN12615000793516). Among these patients, 32 culture-adapted parasites harboring the artemisinin-resistance C580Y K13 mutation were chosen for testing (to assess only piperaquine resistance): 21 from non-recrudescent and 11 from recrudescent patients (Additional file 1) [16].

#### Prospective study

Between May 2014 and February 2015, patients with uncomplicated falciparum malaria were recruited, treated and followed-up for 42 days at district health centres in Rattanakiri, Siem Reap, Stung Treng, and Mondulkiri provinces [16]. Briefly, after obtaining written informed consent from patients or parents/guardians of children, a finger prick blood sample was collected at enrolment for thick/thin blood films and parasite genotyping and a 5 mL venous blood sample was collected into acid-citrate-dextrose tubes for ex vivo PSA. Falciparum malaria was diagnosed by microscopic examination of Giemsa-stained thick/thin blood films and parasitemia was calculated from the number of parasites per 200 white blood cells, assuming a total white cell count of 8000/μL [16]. Patients failing dihydroartemisinin-piperaquine therapy with recurrent *P. falciparum* infection were retreated with artemether plus mefloquine as per national guidelines. Filter-paper blood spots collected on day 0 and day of recurrent parasitemia were used to determine 12 single-nucleotide polymorphisms [11] and classify recurrent infections into reinfections in case of distinct genetic profiles or recrudescent parasites when those profiles were similar. The primary outcome was PCR-corrected *P. falciparum* recrudescence within 42 days (see patient information in Additional file 2). Ethical approvals were obtained from the National Ethical Committee for Health Research of the Cambodian Ministry of Health and the trial was registered at Australian New Zealand Clinical Trials Registry (ACTRN12615000696594).

### In vitro parasite adaptation

Isolates collected on day 0 of enrolment were adapted to in vitro culture and maintained using the following conditions: 2 % hematocrit (O^+^ blood group, blood bank, Phnom Penh, Cambodia) in RPMI 1640 supplemented with 2.5 % decomplemented human plasma (blood bank, Phnom Penh, Cambodia) and 0.5 % Albumax II (Gibco-Life Technologies SAS, France) at 37 °C in a 5 % CO_2_, 5 % O_2_ wet atmosphere [11]. Culture adaptation was considered successful after 3 weeks of culture. The 3D7 reference strain obtained from MR4 was maintained in the same conditions.

### Standard isotopic in vitro susceptibility testing

Piperaquine, mefloquine, dihydroartemisinin and chloroquine were obtained from the WorldWide Antimalarial Resistance Network. In vitro susceptibility of culture-adapted *P. falciparum* parasites was assessed using the 48-h isotopic test monitoring incorporation of [3H]-hypoxanthine (Amersham, Les Ulis, France), as previously described [11], with the 3D7 line as a quality control. Results were expressed as IC_50_ and IC_90_, which values were determined by non-linear regression using the on-line WorldWide Antimalarial Resistance Network IVART software and the on-line ICestimator software (www.antimalarial-icestimator.net), respectively [22].

### Piperaquine survival assays (PSA)

**Fig. 1:**
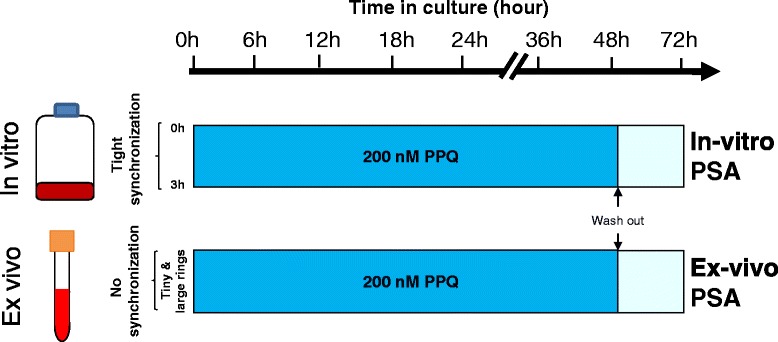
In vitro and ex vivo piperaquine survival assays (PSA). Top: synchronization and timing of 200 nM piperaquine exposure for in vitro PSA performed on culture-adapted *P. falciparum* isolates collected on day 0 from patients subsequently followed-up and presenting or not a late recrudescence. Bottom: timing of 200 nM piperaquine exposure for the ex vivo PSA performed on circulating parasites obtained directly from the blood of patients with falciparum uncomplicated malaria. The ex vivo PSA was performed only on isolates with parasite densities ≥0.1 %. The survival rates were interpretable when the parasite growth rates (parasite density at 72 h/parasite density at 0 h) were >1.5 for the in vitro PSA and >1 for the ex vivo PSA. Dark blue rectangles show culture medium containing 200 nM piperaquine (exposed culture) or 0.5 % lactic acid (non-exposed culture). Light blue rectangles represent complete culture medium without drugs (exposed and non-exposed cultures). PPQ, Piperaquine; PSA, Piperaquine survival assay

The PSA were performed with 0–3-h post-invasion rings from culture-adapted parasites (in vitro PSA) or directly with parasites collected from patients (ex vivo PSA) (Fig. 1). Parasite density and hematocrit levels were adjusted to 0.1–2 % and 2 %, respectively. Parasites were cultivated for 48 h at 37C° under a 5 % CO_2_, 5 % O_2_ wet atmosphere with 200 nM piperaquine tetraphosphate tetrahydrate (exposed culture) or 0.5 % lactic acid (non-exposed culture). After 48 h, cultures were washed once with 12 mL RPMI 1640, resuspended in complete medium (RPMI 1640, 0.5 % Albumax II, 2 % heat-inactivated O+ plasma, 50 μg/mL gentamicin), and cultured for a further 24 h. Thin blood smears were prepared, methanol-fixed and stained with 10 % Giemsa (Merck KGaA, Darmstadt, Germany) for 45 min. The proportion of viable parasites in exposed and non-exposed cultures was evaluated by counting parasites having developed into second-generation rings or trophozoites with normal morphology. For each assay, 20,000 erythrocytes were assessed by two independent microscopists blinded to the clinical data. In case of a difference greater than 20 %, slides were examined by a third microscopist, also blinded to the clinical data. Susceptibility to piperaquine was defined as the median survival rate calculated using the following formula:

$$ \mathrm{P}\mathrm{S}\mathrm{A}\ \mathrm{survival}\ \mathrm{rate}\ \left(\%\right)=\frac{\mathrm{Number}\ \mathrm{o}\mathrm{f}\ \mathrm{viable}\ \mathrm{parasites}\ \mathrm{in}\ \mathrm{exposed}\ \mathrm{culture}}{\mathrm{Number}\ \mathrm{o}\mathrm{f}\ \mathrm{viable}\ \mathrm{parasites}\ \mathrm{in}\ \mathrm{n}\mathrm{o}\mathrm{n}\hbox{-} \mathrm{exposed}\ \mathrm{culture}}\times 100. $$

### Detection of mutations and copy number variation of candidate resistant genes

Following gDNA extraction from the 32 culture-adapted parasites (QIAamp DNA Blood Mini Kit, Qiagen, Valencia, CA), whole-genome sequencing was performed using Illumina paired-reads sequencing [17]. Raw sequence files were filtered using Fqquality tool and the trimmed reads from controlled Fastq files were mapped on the *P. falciparum* 3D7 reference genome with the Burrows-Wheeler Alignment. A pileup file was prepared using Samtools and formatted using in-house software to implement the data into the Wholegenome Data Manager database [17], which was used to align partial or whole genomes and detect mutations or copy number variation in PF3D7_0709000 [23], PF3D7_0523000 [16, 24], PF3D7_0112200 [25], PF3D7_1229100 [26], MAL10:688956 [13], MAL13:1718319 [13], PFE1010w [23], and PFE1085w [23] (Table 1).

**Table 1:** Association between candidate molecular markers (mutations and copy number variation (CNV)) previously associated with piperaquine resistance and in vitro piperaquine survival assay (PSA) phenotypes of 32 culture-adapted isolates collected from patients treated with 3-day courses of dihydroartemisinin-piperaquine in 2012

Gene polymorphism		Piperaquine survival assay (PSA)					
		n	Survival rate (median, IQR)	*P* value^*^	PSA <10 % n = 8	PSA ≥10 % n = 21	*P* value ^**^
***P. falciparum chloroquine resistant transporter gene - Pfcrt (PF3D7_0709000)***							
Alleles	74I/75E/76 T/220S/271E/326S/343L/356 T/371I	7	61.5 (53.6–69.2)	**0.005**	0 (0 %)	7 (33 %)	0.03^***^
	74I/75E/76 T/220S/271E/326S/356T/371I (Dd2 allele)	16	12.8 (0.6–43.7)		8 (100 %)	8 (38 %)	
	74I/75E/76 T/220S/271E/326S/353V/356 T/371I	2	49.2 (37–61.5)		0 (0 %)	2 (9.5 %)	
	74I/75E/76 T/97Y/220S/271E/326S/356T/371I	4	47.1 (40.9–61.2)		0 (0 %)	4 (19 %)	
***P. falciparum multidrug resistance 1*** **gene** ***- Pfmdr-1 (PF3D7_0523000)***							
Alleles	643D	1	70.6	0.16	0 (0 %)	1 (5 %)	0.27
	184 F	16	51.8 (3.5–61.5)		5 (63 %)	11 (52 %)	
	184 F/293D	3	19.3 (9.3–35.4)		1 (12 %)	2 (9.5 %)	
	184 F/1087 L	6	44 (40.1–49.6)		0 (0 %)	6 (29 %)	
	wild-type	3	0.8 (0.5–27.9)		2 (25 %)	1 (5 %)	
CNV (957885 to 962144)	single copy	24	48.1 (38.2–60.1)	**0.001**	3 (38 %)	21 (100 %)	10^−3***^
	multi-copy (184 F, n = 2 and wild-type, n = 3)	5	0.6 (0.4–2.1)		5 (62 %)	0 (0 %)	
***P. falciparum multidrug resistance-associated protein*** **1 -** ***Pfmrp-1 (PF3D7_0112200)***							
Alleles	191Y/437A/785 N/876 V/1007 M	3	0.8 (0.5–83.1)	0.15	2 (25 %)	1 (5 %)	0.02^***^
	191Y/437A/876 V	1	37		0 (0 %)	1 (5 %)	
	191Y/437A/876 V/1390 T	16	50.5 (39.7–60.1)		1 (13 %)	15 (72 %)	
	191Y/437A/876 V/1390 T/1669D	1	0.6		1 (12 %)	0 (0 %)	
	191Y/325S/437A	8	22.9 (0.6–47.1)		4 (50 %)	4 (19 %)	
***P. falciparum multidrug resistance-associated protein*** **2 -** ***Pfmrp-2 (PF3D7_1229100)***							
Alleles	199 V/295R/593D/714I/1527 T/1531I	1	0.7	0.07	1 (12.5 %)	0 (0 %)	0.003^***^
	199 V/622D/646D/714I/1188 N/1527 T/1531I	2	3.5 (0.6–6.4)		2 (25 %)	0 (0 %)	
	199 V/646D/714I/1176 N/1188 N/1527 T/1531I	1	0.8		1 (12.5 %)	0 (0 %)	
	199 V/646D/714I/1188 N/1527 T/1531I	10	58.7 (29.0–62.1)		2 (25 %)	8 (35 %)	
	199 V/646D/714I/1527 T/1531I	16	41.7 (38.2–51.6)		1 (12.5 %)	15 (65 %)	
	199 V/646D/714I/964D/970 N/1527 T/1531I	1	0.3		1 (12.5 %)	0 (0 %)	
***MAL10:688956*** **(Chr10)**							
Alleles	3D7 allele type (T)	10	22.9 (0.6–51.8)	0.20	5 (62.5 %)	5 (24 %)	0.08
	V1/S mutant-type (A)	19	46.7 (31–58.2)		3 (37.5 %)	16 (76 %)	
***MAL13:1718319*** **(Chr13)**							
Alleles	3D7 allele type (A)	1	0.3	–	1 (12.5 %)	0 (0 %)	0.27
	V1/S mutant-type (T)	28	41.6 (12.8–57.7)		7 (87.5 %)	21 (100 %)	
***PFE1010w*** **(Chr5, 831614 to 834340)**							
CNV	single copy	29	–	–	8 (100 %)	21 (100 %)	–
	multi-copy	0	–		0 (0 %)	0 (0 %)	–
***PFE1085w*** **(Chr5, 882373 to 884898)**							
CNV	single copy	29	–	–	8 (100 %)	21 (100 %)	–
	multi-copy	0	–		0 (0 %)	0 (0 %)	–

Pfcrt Dd2, MAL10:688956 and MAL13:1718319 allele types are defined based on sequences available at plasmoDB.org

^*^Calculated by the Mann-Whitney U or Kruskal-Wallis (H-test) tests

^**^Calculated by the Fisher exact or χ^2^ tests

^***^Significant *P* values are shown in bold font

DNA from day 0 blood samples (2014-propective study) was used to genotype the K13-propeller domain (PF3D7_1343700) and measure *P. falciparum multidrug resistance 1* copy number, as previously described [16].

### Statistical analysis

Data were analyzed with Microsoft Excel and MedCalc version 12 (Mariakerke, Belgium). Quantitative and qualitative data were expressed as median (interquartile range, IQR) or proportion (%), respectively. The Mann-Whitney U or the Kruskal-Wallis (H-test) tests were used for non-parametric comparisons. For categorical variables, proportions were examined by χ^2^ or by Fisher exact tests. Relative risks were estimated using the Mantel-Haenszel test. The cumulative risk of failure at day 42 was assessed by survival analysis with the Kaplan-Meier method. Treatment outcome between patients harboring mutant or wild-type K13 parasites and PSA ex vivo survival rate < or ≥10 % at day 0 were compared using the Mantel-Haenszel log rank test and hazard ratio (HR). Two sided *P* values of <0.05 were considered statistically significant.

## Results

### In vitro phenotype of piperaquine resistance: 2012 retrospective analysis

Investigations of various risk factors associated with dihydroartemisinin-piperaquine failure in 32 artemisinin-resistant culture-adapted parasites collected at enrolment showed that the only significant parameter was the median mefloquine IC_50_, which was significantly higher in isolates of non-recrudescent compared to recrudescent patients (32.2 nM, IQR: 19.4–39.8 nM vs. 19.7 nM, IQR: 15.5–22.2 nM, respectively, *P* = 0.03) (Table 2).

**Table 2:** Patient and parasitological characteristics of 32 culture-adapted isolates and their association with dihydroartemisinin-piperaquine treatment outcome at day 42 in Cambodian patients, Cambodia (2012 retrospective study)

Risk factors		All patients n = 32	Non-recrudescent patients n = 21	Recrudescent patients n = 11	*P* value
Patient					
Age, years (median, IQR)		19 (16.5–23)	19 (17.2–22.2)	19 (14.2–23.5)	0.67^*^
Sex, male (n, %)		21 (66 %)	13 (62 %)	8 (73 %)	0.70^**^
Weight, kg (median, IQR)		49.5 (41.5–55.0)	50 (42.7–55.2)	45.0 (40.2–54.7)	0.49^*^
Axillary temperature, °C (median, IQR)		38.5 (38.1–39.5)	38.5 (38.4–39.5)	38.5 (38.1–39.4)	0.87^*^
Dihydroartemisinin dose, mg/kg/day (median, IQR)		2.4 (2.2–2.7)	2.4 (2.2–2.6)	2.4 (2.2–2.7)	0.53^*^
Piperaquine dose, mg/kg/day (median, IQR)		19.2 (17.5–21.3)	18.8 (17.4–21.0)	19.4 (17.6–21.7)	0.47^*^
Target dose ≥2/16 mg/kg/d DHA/PPQ (n, %)		29 (91 %)	18 (86 %)	11 (100 %)	0.53^**^
Day 7 plasma piperaquine concentration, ng/mL (median, IQR)		41.8 (31.6–58.5)	40.1 (30.4–56.7)	47.5 (32.7–60.4)	0.53^*^
Day 3 parasite positive (n, %)		10 (31 %)	6 (29 %)	4 (36 %)	0.70^**^
Parasite					
Day 0 N° parasites per μL (median, IQR)		15,879 (5,961–64,291)	13,936 (6,960–47,097)	28,455 (5,250–81,063)	0.59^*^
Presence of K13 C580Y allele (n, %)		32 (100 %)	21 (100 %)	11 (100 %)	1^**^
*Pfmdr1* copy number (median, range)		1 (1–3)	1 (1–3)	1 (1)	0.11^*^
Chloroquine IC_50_ at D0 (nM)	Interpretable IC_50_ (n, %)	31 (97 %)	20 (95 %)	11 (100 %)	1^**^
	median, IQR	183 (94–308)	199 (72–306)	150 (126–389)	0.87^*^
Piperaquine IC_50_ at D0 (nM)	Interpretable IC_50_ (n, %)	12 (37 %)	10 (48 %)	2 (18 %)	0.14^**^
	median, IQR	42.9 (22.4–52.7)	40.3 (7.6–52.1)	55.6	0.39^*^
Piperaquine IC_90_ at D0 (nM)	Interpretable IC_90_ (n, %)	12 (37 %)	10 (48 %)	2 (18 %)	0.14^**^
	median, IQR	81.7 (58.1–119.8)	72.2 (54.3–96.0)	132.6	0.06^*^
Mefloquine IC_50_ at D0 (nM)	median, IQR	42.9 (22.4–52.7)	40.3 (7.6–52.1)	55.6	0.39^*^
	median, IQR	24.4 (18.4–34.3)	32.2 (19.4–39.8)	19.7 (15.5–22.2)	0.03^*,***^
DHA IC_50_ at D0 (nM)	Interpretable IC_50_ (n, %)	31 (97 %)	20 (95 %)	11 (100 %)	1^**^
	median, IQR	0.86 (0.61–1.58)	0.88 (0.63–1.86)	0.83 (0.61–1.02)	0.30^*^
Survival rate (RSA^0–3h^) (n, %)	Interpretable (n, %)	32 (100 %)	21 (100 %)	11 (100 %)	1^**^
	median, IQR	13.2 (9.9–18.1)	12.3 (10.0–15.2)	15.2 (8.1–20.9)	0.54^*^

^*^Calculated by the Mann-Whitney U test

^**^Calculated by Fisher exact test

^***^Significant *P* values

**Fig. 2:**
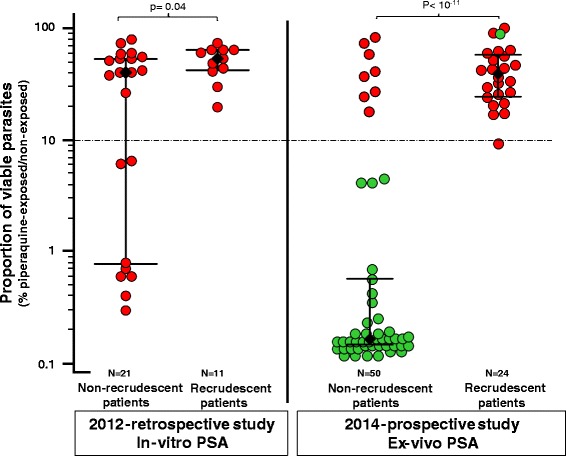
Association between clinical dihydroartemisinin-piperaquine outcome and in vitro and ex vivo piperaquine survival assay (PSA) survival rates. In vitro and ex vivo PSAs were done with 0–3 h post-invasion rings from culture-adapted parasites isolated in 2012–2013 or parasites directly collected from patients with malaria in Rattanakiri, Siem Reap, Stung Treng, and Mondulkiri in 2014, respectively. Results from the in vitro and ex vivo PSAs are expressed as the proportion of viable parasites in the exposed or non-exposed cultures (Fig. 1). Isolates (collected on day 0) are dichotomized according to the clinical outcome of infection in patients enrolled and treated with a 3-day course of dihydroartemisinin-piperaquine (non-recrudescence or recrudescence of *P. falciparum* infections within 42 days, after PCR-correction). The median of the proportion of viable parasites was significantly higher in isolates from subsequently recrudescent than non-recrudescent patients (in vitro PSAs 51.9 % vs. 34.4 %, respectively, *P* = 0.04; ex vivo PSA: 39.2 % vs. 0.17 %, respectively, *P* <1 × 10^–11^). Each circle represents a *P. falciparum* isolate. Red and green colors refer to K13 mutant alleles (C580Y or Y493H) and K13 wild-type alleles, respectively. The black diamonds, the horizontal lines and I bars represent the medians and interquartile ranges. The dotted grey line represents the 10 % survival rate cut-off that distinguishes piperaquine-resistant (≥10 %) from piperaquine-sensitive (<10 %) parasites in PSAs

Overall, we observed a lower frequency of interpretable curves for piperaquine (12/32, 37 %) compared to the other drugs tested (28/32; 87 % for mefloquine and 31/32, 97 % for chloroquine and dihydroartemisinin). A paradoxical increase of incorporation at high drug concentrations (≥100–200 nM piperaquine) was repeatedly observed (Additional file 3), and this was more frequent in isolates from recrudescent (9/11, 82 %) than from non-recrudescent patients (11/21, 52 %). In contrast, every culture-adapted isolate gave interpretable in vitro PSA data and 83 % (19/23) of the isolates with a PSA survival rate ≥10 % had an unreliable concentration-response curve for piperaquine. Median survival rates were higher in day 0 isolates from recrudescent (51.9 %, IQR: 40.7–61.5 %) than non-recrudescent patients (34.4 %, IQR: 0.8–52.2 %, *P* = 0.04, Table 2). All day 0 culture-adapted isolates from recrudescent patients had a PSA survival rate ≥10 %, whereas survival rates from non-recrudescent patients ranged from 0.3 % to 77.4 % (Fig. 2).

### Ex vivo PSA: 2014 prospective study

In a prospective study conducted in 2014, 178 patients presenting to district health centres with uncomplicated falciparum malaria were enrolled, administered standard 3-day dihydroartemisinin-piperaquine treatment and followed-up to day 42 or day of failure [16]. Detection of K13-propeller mutations and ex vivo PSA survival rates of day 0 isolates were achieved for 74 patients (Additional file 4): 50 patients were classified as non-recrudescent and 24 patients as recrudescent, with a mean time to recrudescence of 28 days (IQR: 21–32.5 days).

Recrudescent infections were strongly associated with day 0 parasites presenting high survival rates in the ex vivo PSA (median = 39.2 %, IQR: 24.5–57.6 %) contrasting with parasites from non-recrudescent patients that had uniformly low survival (median = 0.17 %, IQR: 0.15–0.59 %, *P* <1 × 10^−11^) (Fig. 2). All day 0 blood samples from recrudescent patients had PSA survival rates ≥10 %. Recrudescence was also associated with presence of a mutant K13 allele (C580Y or Y493H) on day 0 (8/50, 16 % in non-recrudescent patients vs. 23/24, 96 % in recrudescent patients, *P* <1 × 10^−10^). Thus, artemisinin resistance (defined as presence of a mutant K13 allele) was strongly associated with piperaquine resistance (defined as PSA survival rate ≥10 %; *P* <1 × 10^−14^) (Fig. 2 and Additional file 2).

**Fig. 3:**
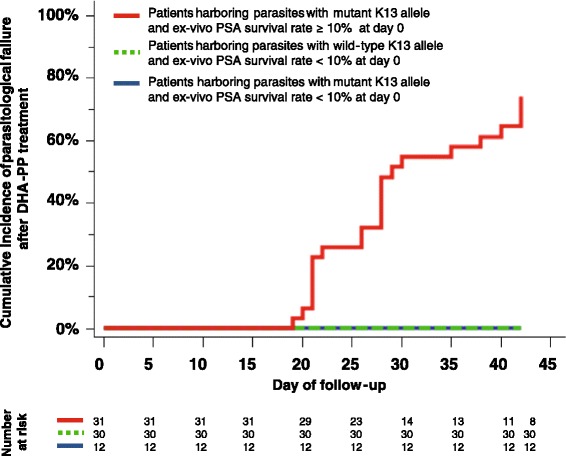
Cumulative incidence of clinical failure within 42 days (after PCR-correction) in patients treated with a 3-day dihydroartemisinin-piperaquine course according to K13 allele (wild-type or mutant) and ex vivo piperaquine survival assay (PSA) survival rates of day 0 parasites. The cumulative incidence of clinical failure was significantly higher in patients infected on day 0 by isolates carrying a mutant K13 allele and presenting a PSA survival rate ≥10 % (*P* <1 × 10^–10^, log rank test, Hazard Ratio = 14.3, 95 % CI, 4.6–44.6, Fig. 3). The survival proportion at day 42 for those patients was estimated 25.8 % (SD = 7.9 %)

The cumulative incidence of parasitological failure after the 3-day dihydroartemisinin-piperaquine treatment was significantly higher in patients infected by mutant K13 parasites with a PSA survival rate ≥10 % (*P* <1 × 10^−10^, log rank test, Hazard Ratio = 14.3, 95 % CI, 4.6–44.6; Fig. 3). These patients had a 32-fold higher risk of recrudescence (95 % CI, 4.5–224; *P* = 0.0005); only 25.8 % (SD = 7.9 %) of these patients remained without parasites at day 42 after treatment.

Of note, as observed previously for artemisinin resistance [11], data from the prospective study show a progressive decrease in piperaquine-resistant *P. falciparum* parasites from Western to Eastern Cambodia: 88.2 % (15/17) in Siem Reap, 61.5 % (8/13) in Stung Treng to 25.0 % (4/16) and 21.4 % (6/28) in Mondulkiri and Rattanakiri, respectively.

### Candidate molecular markers associated with piperaquine resistance

The 32 C580Y-K13 culture-adapted parasites were analyzed for possible association between candidate molecular markers and in vitro PSA survival rates (Table 1). Of eight candidate genes tested [13, 16, 23–26], specific mutations of *Pfcrt* and copy number variation of *Pfmdr1* were highly associated with piperaquine resistance (Additional file 5). The isolates containing parasites with a variant of the Dd2 *Pfcrt* allele carrying either 97Y, 343 L or 353 V had higher median survival rates compared to those harboring the Dd2 allele. *Pfmdr-1* single copy parasites had higher median survival rates than *Pfmdr-1* multi-copy parasites (48.1 % vs. 0.6 %, *P* <1 × 10^–3^; Table 1).

## Discussion

Declining efficacy of ACTs and, more specifically, of dihydroartemisinin-piperaquine, can jeopardize the gains obtained during the last decade in controlling malaria in Cambodia. The proportion of patients who experience late treatment failure with dihydroartemisinin-piperaquine is on the increase and, worryingly, this trend appears to be spreading eastwards in the country. Artemisinin resistance accounts for slower clearance rates in the first 3 days of treatment, but late recrudescence is believed to reflect incomplete efficacy of the long half-life partner drug. Although significant progress has been made recently in the detection of artemisinin-resistant *P. falciparum* parasites [11, 17, 19], tools to detect piperaquine resistance more rapidly than the 42-day post-treatment outcome are urgently needed. The work reported here fills this important gap in providing a robust in vitro assay clearly differentiating resistant isolates from susceptible ones.

IC_50_ data from standard in vitro assays have proved so far to be inadequate to assess piperaquine resistance; IC_50_ values have not distinguished recrudescent and non-recrudescent isolates in patients treated with dihydroartemisinin-piperaquine [12, 13, 15, 16, 27]. The poor performance of the standard assays in this regard is possibly due to the relatively high frequency of non-interpretable curves observed frequently in assays of piperaquine resistant isolates. Indeed, most of the isolates collected from recrudescent patients (9/11) studied here gave non-interpretable curves (Additional file 3), although all had conventional response curves to the other drugs tested. These anomalous curves presented a paradoxical increase of [3H]-hypoxanthine incorporation at piperaquine concentrations above 100–200 nM, the physiological concentration of piperaquine observed in blood in patients treated with a standard 3-day course of dihydroartemisinin-piperaquine during the first 3 days [21]. Several factors might contribute to such atypical profiles, reported previously for several ACT partner drugs, including altered transcriptional responses, increased protein production or nucleic acid precursor uptake for drugs ineffective on ring-stages, plate edge effects or mixed-clone infections [22]. This last factor can be excluded here as we used culture-adapted single clone lines as well as the insolubility of the piperaquine powder at high concentrations (>100 nM) in drug test wells, as presented in Additional file 6. Our data rather suggest that the paradoxical profiles might reflect an inducible mechanism of piperaquine resistance triggered at physiological concentrations (~200 nM). Inducible responses were avoided in the PSA by assessing the viability of the parasites over a 24 h period after the 48 h incubation. Importantly, non-interpretable curves were significantly more frequent for isolates with in vitro PSA survival rates ≥10 % (83 % vs. 0 %, *P* <1 × 10^−4^; Additional file 1). This cannot be reliably used as a phenotype proxy of piperaquine resistance as the effect cannot be differentiated from failed assays. To overcome the limitations of the current assays and obtain a robust assessment of piperaquine resistance, we developed the PSA based on the detection of viable parasites after exposure to 200 nM for 48 h. The PSA was designed to mimic in vivo exposure of *P. falciparum* parasites to physiological concentrations of piperaquine (200 nM) for 48 h (all parasite stages – from 0–3 h early ring stages to 48 h schizonts – were then exposed to piperaquine, as the piperaquine half-life is ~9 days after a standard cure in treated patients, to globally assess the parasite susceptibility over its entire life cycle). The assay was not designed to investigate the piperaquine susceptibility to different parasite stages (by exposing the parasite stages to shorter pulses). These experiments deserve to be performed in future studies to comprehensively decipher the mechanism of action/resistance of *P. falciparum* to piperaquine. Despite these limitations, the PSA highlights, for the first time, that survival rates from the ex vivo testing strongly correlate with the clinical outcome of 3-day dihydroartemisinin-piperaquine treatment. All samples isolated at day 0 from recrudescent infections had a PSA survival rate ≥10 %, a cut-off value that can be used to define resistance to piperaquine. The ex vivo PSA remarkably captured the survival capacity/potential of day 0 parasites exposed to piperaquine, which became overt in patients only several weeks later. This was probably facilitated by the low complexity of day 0 infections, which in the large majority consisted of single parasite clones.

In vitro PSA testings carried out with isolates harboring the C580Y K13 mutation showed that resistance to piperaquine is not directly related to K13 polymorphism as 8 of 21 C580Y, artemisinin-resistant parasites isolated from non-recrudescent infections were susceptible to piperaquine (PSA <10 %; Fig. 2). However, the prospective study showed that essentially all parasites presenting a survival rate ≥10 % also carried a mutant K13 locus. This likely reflects the fact that, in patients, piperaquine resistance was selected from parasites that were already artemisinin-resistant, necessitating survival first to the fast acting drug (three short-pulses of dihydroartemisinin) and afterwards to the long-acting partner drug (piperaquine). The association with artemisinin resistance reflects this two-step selection process rather than being causal. In Western Cambodia, the extremely limited genetic diversity of the parasite populations means that almost all parasites carry K13 polymorphisms [17, 28, 29] and the selection for resistance to piperaquine was presumably correspondingly strong. Piperaquine resistance was also associated with other genetic polymorphisms. We confirm that single-copy *Pfmdr-1* (and consequently low mefloquine IC_50_) is associated with piperaquine resistance. All piperaquine-resistant isolates (in vitro PSA survival rates ≥10 %) had a single *Pfmdr-1* copy (median PSA survival rate = 48 % vs. 0.6 % in *Pfmdr-1* multiple copy isolates, *P* = 0.0001), but the reverse was not true, as isolates with a single copy of *Pfmdr-1* were not all piperaquine-resistant (Additional file 5). This finding strongly supports the recent recommendation of the Cambodian National malaria control programs for using artesunate plus mefloquine as first-line treatment in provinces where dihydroartemisinin-piperaquine failure rates are above 10 %. We also found a possible association with three independent *Pfcrt* mutations on the Dd2 genetic background (Y97, L343, and V353). Whether these associations reflect the structure of Cambodian parasite populations stemming from recent bottlenecks [28, 29] or a direct contribution to the phenotype remains to be investigated using genome-wide association studies and gene editing. The PSA will be particularly useful for such studies.

## Conclusion

The data presented here demonstrate that the ex vivo PSA is a convenient method for monitoring piperaquine resistance in the field, especially in areas of artemisinin resistance such as Vietnam and Myanmar where dihydroartemisinin-piperaquine is the recommended first line treatment of uncomplicated falciparum malaria. Ex vivo PSA combined with K13 genotyping informing on both artemisinin and piperaquine resistance has the potential to provide timely evidence at the country level and complement the therapeutic efficacy studies to inform national malaria control programs and policymakers about existing or emerging risks of artemisinin and piperaquine resistance.

### Consent to publish

A written informed consent was obtained from any enrolled patients for publication. A copy of the written consent is available for review by the Editor of this journal.

## Additional files

Additional file 1:
**Patient information and corresponding data from in vitro piperaquine survival assays performed on 32 culture-adapted** ***P. falciparum*** **isolates from Cambodia collected on day 0 in 2012 from malaria patients treated with dihydroartemisinin-piperaquine.** (PDF 94 kb) Additional file 2:
**Patient information and corresponding data from ex vivo piperaquine survival assays performed on** ***P. falciparum*** **isolates from Rattanakiri, Siem Reap, Stung Treng and Mondulkiri provinces in 2014.** (PDF 77 kb) Additional file 3:
**Examples of interpretable piperaquine concentration-inhibition curves (Panel A) and non-interpretable piperaquine concentration-inhibition curves due to a paradoxical increase of apparent growth at high drug concentrations (Panel B: curves do not fit the data; Panel C: core criteria of curves are not acceptable).** (PDF 40 kb) Additional file 4:
**Flow chart of patients included in the final analysis of the 2014 retrospective study.** Cambodia 2014 and isolates used in ex vivo assays. (PDF 52 kb) Additional file 5:
**In vitro piperaquine survival assay survival rate distribution in 32 culture-adapted** ***P. falciparum*** **isolates from Cambodia in 2012 according to mutations or copy number variation in candidate genes significantly associated with piperaquine resistance (see Table** **1** **).** Panel A: mutations of *P. falciparum chloroquine resistant transporter* gene (*Pfcrt*); Panel B: copy number variation of *P. falciparum multidrug resistance 1* gene (*Pfmdr-1*). (PDF 85 kb) Additional file 6:
**Results of the piperaquine concentration measurements in culture supernatants of different wells in plate used for the classical isotopic assay, containing increasing piperaquine concentrations (from 1.91 nM in PPQ11 to 2000 nM in PPQ1).** (DOCX 12 kb)

## Abbreviations

ACT: Artemisinin combination therapy
IC: Inhibitory concentration
PSA: Piperaquine survival assay
