# Supplementary material for: Plasmodium falciparum dihydroartemisinin-piperaquine failures in Cambodia are associated with mutant K13 parasites presenting high survival rates in novel piperaquine in vitro assays: retrospective and prospective investigations
Source: BMC Med. 2015 Dec 22;13:305. doi: 10.1186/s12916-015-0539-5 (PMC4688949; doi:10.1186/s12916-015-0539-5)
Supplement: Additional file 1: — Patient information and corresponding data from in vitro piperaquine survival assays performed on 32 culture-adapted P. falciparum isolates from Cambodia collected on day 0 in 2012 from malaria patients treated with dihydroartemisinin-piperaquine. (PDF 94 kb) [file 12916_2015_539_MOESM1_ESM.pdf]

Patient ID	Site (province)	Age (years)	Sex	Parasitemia at day 0 (/mm^3)	Clinical outcome at day 42 [1]	K13 allele [2]	Day of recrudescence	Parasitemia at day of recrudescence (/mm^3)	RSA survival rate (%) [3]	PSA survival rate (%) [4]	IC_{50} PP (nM)	IC_{50} CQ (nM)	IC_{50} DHA (nM)	IC_{50} MQ (nM)
6246	Kampong Speu	18	M	100.804	Non recrudescent	C580Y	-	-	15.2	37.0	NI	61	0.48	39.8
6293	Battambang	19	M	13.936	Non recrudescent	C580Y	-	-	11.5	39.4	NI	185	0.45	35.5
6391	Battambang	18	M	47.070	Non recrudescent	C580Y	-	-	12.3	39.4	NI	668	0.83	7.5
6218	Battambang	19	M	47.179	Non recrudescent	C580Y	-	-	14.7	40.8	NI	39	0.25	11.3
6443	Battambang	20	F	114,500	Non recrudescent	C580Y	-	-	7.3	49.6	NI	277	2.13	NI
6430	Battambang	44	F	4,010	Non recrudescent	C580Y	-	-	10.2	51.4	NI	338	0.54	NI
6429	Pursat	18	M	7,943	Non recrudescent	C580Y	-	-	22.8	51.8	NI	387	0.74	28.3
6394	Battambang	31	F	10,945	Non recrudescent	C580Y	-	-	5.5	56.8	NI	NA	NA	NI
6408	Battambang	19	F	1,572	Non recrudescent	C580Y	-	-	9.6	58.7	NI	183	1.68	24.0
6411	Battambang	11	F	23,114	Non recrudescent	C580Y	-	-	14.0	71.6	NI	308	0.89	28.9
6427	Battambang	19	F	9,117	Non recrudescent	C580Y	-	-	22.8	77.4	NI	358	1.92	15.4
6273	Kampong Speu	21	M	17,822	Non recrudescent	C580Y	-	-	14.5	0.3	6.1	181	2.49	55.1
6337	Kampong Speu	26	M	2,064	Non recrudescent	C580Y	-	-	19.3	0.4	53.3	215	1.79	91.4
6403	Pursat	12	M	55,663	Non recrudescent	C580Y	-	-	7.3	0.6	7.1	159	1.64	33.1
6267	Kampong Speu	15	M	13,628	Non recrudescent	C580Y	-	-	11.1	0.6	7.6	289	2.14	117.4
6349	Kampong Thom	18	M	40,493	Non recrudescent	C580Y	-	-	13.7	0.7	43.4	56	2.42	97.8
6237	Kampong Thom	19	M	10,348	Non recrudescent	C580Y	-	-	11.5	0.8	37.2	18	0.48	32.1
6410	Battambang	29	F	126,701	Non recrudescent	C580Y	-	-	15.4	6.0	104.3	303	0.88	18.4
6369	Pursat	10	M	38,854	Non recrudescent	C580Y	-	-	10.5	6.4	52.1	213	0.83	32.2
6341	Pursat	26	F	1,662	Non recrudescent	C580Y	-	-	8.4	25.8	37.2	84	0.89	36.3
6286	Battambang	12	M	401	Non recrudescent	C580Y	-	-	17.4	53.5	44.2	57	0.72	19.3
6395	Battambang	11	M	83,612	Recrudescent	C580Y	35	4,458	12.8	19.3	NI	131	1.18	15.4
6280	Battambang	18	M	1,594	Recrudescent	C580Y	21	79	30.6	29.0	NI	124	0.48	24.8
6272	Battambang	45	M	118,252	Recrudescent	C580Y	35	27,421	11.0	40.1	NI	440	0.83	11.6
6302	Battambang	20	M	4,539	Recrudescent	C580Y	35	597	7.2	42.5	NI	148	0.71	32.2
6229	Battambang	19	M	2,640	Recrudescent	C580Y	21	492	21.1	46.7	NI	552	1.01	19.4
6224	Pursat	22	M	7,938	Recrudescent	C580Y	14	NA	22.0	61.5	NI	33	0.23	19.9
6431	Battambang	13	M	73,418	Recrudescent	C580Y	21	157	4.9	61.5	NI	482	1.03	NI
6320	Battambang	24	M	28,455	Recrudescent	C580Y	42	5,426	18.9	62.1	NI	232	1.43	22.2
6261	Pursat	12	F	72,919	Recrudescent	C580Y	28	NA	20.4	70.6	NI	166	0.81	21.7
6365	Battambang	41	F	7,384	Recrudescent	C580Y	28	18,817	5.5	51.9	42.4	150	0.58	9.8
6219	Battambang	19	F	143,700	Recrudescent	C580Y	21	10,432	15.2	58.7	68.8	78	0.86	18.3

[1] For each patient, the clinical outcome was evaluated at day 42, after PCR-correction (distinction between reinfection and recrudescent in case of *P falciparum* recurrent infections) according to the 2009 WHO protocol.

[2] Mutation in the propeller domain of the k13 gene was assessed by nested PCR and sequencing (see ref 18).

[3] RSA$^{0\text{-}3h}$ was performed as previously described (see ref 10). In-vitro RSA assays were considered as interpretable if the growth rate in non-exposed culture (parasitemia at 72h/parasitemia at 0h) was ≥1.5. The limit of detection of viable parasites was estimated 0.1% parasitemia (number of red blood cells counted by each microscopist = 20,000 and average parasitemia in non-exposed = 5%).

[4] PSA survival rates (%) are expressed as the median of the survival rates (expressed as percent of viable parasites). In-vitro PSA assays were considered as interpretable if the growth rate in non-exposed culture (parasitemia at 72h/parasitemia at 0h) were >1.5. The limit of detection of viable parasites was estimated 0.1% parasitemia (number of red blood cells counted by each microscopist = 20,000 and average parasitemia in non-exposed = 5%).

NI : Non-interpretable curves ; NA : Not available data.

IC_{50} PP: Inhibitory concentration 50% for piperaquine; IC_{50} CQ: Inhibitory concentration 50% for chloroquine; IC_{50} DHA: Inhibitory concentration 50% for dihydroartemisinin; IC_{50} MQ: Inhibitory concentration 50% for mefloquine.
