# Supplementary material for: Plasmodium falciparum dihydroartemisinin-piperaquine failures in Cambodia are associated with mutant K13 parasites presenting high survival rates in novel piperaquine in vitro assays: retrospective and prospective investigations
Source: BMC Med. 2015 Dec 22;13:305. doi: 10.1186/s12916-015-0539-5 (PMC4688949; doi:10.1186/s12916-015-0539-5)
Supplement: Additional file 2: — Patient information and corresponding data from ex vivo piperaquine survival assays performed on P. falciparum isolates from Rattanakiri, Siem Reap, Stung Treng and Mondulkiri provinces in 2014. (PDF 77 kb) [file 12916_2015_539_MOESM2_ESM.pdf]

Patient ID	Site	Age (years)	Sex	PCR_corrected clinical outcome at day 42 [1]	Day of recrudescence	Day 0 isolate K13 allele [2]	Ex-vivo PSA survival rate (%) [3]	K13 and ex-vivo PSA classification [4]
6960	Mondulkiri	37	M	Non recrudescent	-	C580Y	0.12	Group 1
6904	Mondulkiri	20	M	Non recrudescent	-	Wild type 3D7	0.12	Group 0
6978	Mondulkiri	33	M	Non recrudescent	-	Wild type 3D7	0.12	Group 0
6885	Ratanakiri	32	F	Non recrudescent	-	Wild type 3D7	0.12	Group 0
6973	Mondulkiri	30	M	Non recrudescent	-	C580Y	0.13	Group 1
6880	Ratanakiri	29	F	Non recrudescent	-	Wild type 3D7	0.13	Group 0
6845	Rattanakiri	5	F	Non recrudescent	-	Wild type 3D7	0.14	Group 0
6847	Rattanakiri	25	M	Non recrudescent	-	Wild type 3D7	0.14	Group 0
6857	Rattanakiri	20	F	Non recrudescent	-	Wild type 3D7	0.14	Group 0
6952	Stungtreng	22	M	Non recrudescent	-	Wild type 3D7	0.14	Group 0
6866	Rattanakiri	8	M	Non recrudescent	-	Wild type 3D7	0.15	Group 0
6881	Rattanakiri	28	M	Non recrudescent	-	Wild type 3D7	0.15	Group 0
6853	Rattanakiri	3	M	Non recrudescent	-	Wild type 3D7	0.15	Group 0
6888	Rattanakiri	45	M	Non recrudescent	-	Wild type 3D7	0.15	Group 0
6868	Rattanakiri	20	F	Non recrudescent	-	Wild type 3D7	0.15	Group 0
6878	Rattanakiri	29	M	Non recrudescent	-	Wild type 3D7	0.15	Group 0
6926	Stungtreng	24	M	Non recrudescent	-	Wild type 3D7	0.15	Group 0
6879	Rattanakiri	12	M	Non recrudescent	-	Wild type 3D7	0.16	Group 0
6883	Rattanakiri	18	M	Non recrudescent	-	Wild type 3D7	0.16	Group 0
6831	Rattanakiri	55	M	Non recrudescent	-	Wild type 3D7	0.16	Group 0
6886	Rattanakiri	11	M	Non recrudescent	-	Wild type 3D7	0.16	Group 0
6843	Rattanakiri	4	M	Non recrudescent	-	Wild type 3D7	0.16	Group 0
6903	Stungtreng	13	M	Non recrudescent	-	Wild type 3D7	0.16	Group 0
6930	Mondulkiri	26	M	Non recrudescent	-	C580Y	0.17	Group 1
6956	Mondulkiri	20	M	Non recrudescent	-	Wild type 3D7	0.17	Group 0
6884	Rattanakiri	16	F	Non recrudescent	-	Wild type 3D7	0.17	Group 0
6867	Rattanakiri	15	F	Non recrudescent	-	Wild type 3D7	0.17	Group 0
6862	Rattanakiri	23	M	Non recrudescent	-	Wild type 3D7	0.17	Group 0
6842	Rattanakiri	18	F	Non recrudescent	-	Wild type 3D7	0.18	Group 0

6936	Mondulkiri	24	M	Non recrudescent	-	C580Y	0.19	Group 1
6836	Rattanakiri	11	F	Non recrudescent	-	Wild type 3D7	0.19	Group 0
6933	Stungtreng	57	M	Non recrudescent	-	Wild type 3D7	0.19	Group 0
6875	Rattanakiri	13	F	Non recrudescent	-	Wild type 3D7	0.20	Group 0
7018	Mondulkiri	34	M	Non recrudescent	-	Wild type 3D7	0.24	Group 0
6931	Mondulkiri	15	M	Non recrudescent	-	C580Y	0.26	Group 1
7002	Mondulkiri	11	F	Non recrudescent	-	C580Y	0.36	Group 1
6950	Mondulkiri	26	M	Non recrudescent	-	C580Y	0.44	Group 1
6949	Mondulkiri	50	M	Non recrudescent	-	C580Y	0.59	Group 1
6945	Siemreap	32	M	Non recrudescent	-	C580Y	0.72	Group 1
6944	Siemreap	31	M	Non recrudescent	-	C580Y	4.28	Group 1
6858	Rattanakiri	7	M	Non recrudescent	-	C580Y	4.31	Group 1
6967	Stungtreng	25	M	Non recrudescent	-	C580Y	4.72	Group 1
6928	Siemreap	33	M	Non recrudescent	-	C580Y	18.96	Group 2
6998	Siemreap	23	M	Non recrudescent	-	Y493H	25.73	Group 2
6927	Siemreap	37	M	Non recrudescent	-	C580Y	28.56	Group 2
7025	Siemreap	37	M	Non recrudescent	-	C580Y	38.98	Group 2
6990	Siemreap	40	M	Non recrudescent	-	C580Y	43.67	Group 2
6882	Rattanakiri	20	M	Non recrudescent	-	C580Y	61.60	Group 2
6985	Stungtreng	14	M	Non recrudescent	-	C580Y	77.25	Group 2
6905	Stungtreng	26	M	Non recrudescent	-	C580Y	88.17	Group 2
6953	Mondulkiri	17	M	Recrudescent	29	C580Y	9.17	Group 2
6961	Siemreap	16	M	Recrudescent	21	C580Y	16.65	Group 2
6859	Rattanakiri	45	M	Recrudescent	19	C580Y	17.11	Group 2
6979	Mondulkiri	18	M	Recrudescent	38	C580Y	20.34	Group 2
6910	Stungtreng	17	M	Recrudescent	21	C580Y	20.93	Group 2
6993	Siemreap	22	M	Recrudescent	21	C580Y	23.85	Group 2
6929	Mondulkiri	21	M	Recrudescent	26	C580Y	25.20	Group 2
6977	Stungtreng	60	M	Recrudescent	28	C580Y	26.46	Group 2
7001	Siemreap	20	M	Recrudescent	20	C580Y	29.01	Group 2
6913	Siemreap	19	M	Recrudescent	35	C580Y	31.79	Group 2
6946	Stungtreng	39	M	Recrudescent	28	C580Y	33.07	Group 2

7004	Stungtreng	14	M	Recrudescent	28	C580Y	36.45	Group 2
6971	Siemreap	22	M	Recrudescent	26	C580Y	41.96	Group 2
7000	Siemreap	34	M	Recrudescent	42	C580Y	42.34	Group 2
6943	Stungtreng	52	M	Recrudescent	28	C580Y	43.29	Group 2
6834	Rattanakiri	14	M	Recrudescent	22	C580Y	46.79	Group 2
6901	Stungtreng	15	M	Recrudescent	28	C580Y	51.73	Group 2
6863	Rattanakiri	18	M	Recrudescent	40	C580Y	55.56	Group 2
6994	Mondulkiri	28	M	Recrudescent	30	C580Y	59.67	Group 2
6907	Siemreap	22	M	Recrudescent	21	C580Y	61.51	Group 2
6887	Rattanakiri	45	M	Recrudescent	42	C580Y	62.86	Group 2
6968	Siemreap	48	M	Recrudescent	42	C580Y	90.03	Group 2
6914[5]	Siemreap	23	M	Recrudescent	28	Wild type 3D7	102.36	
6996	Siemreap	32	M	Recrudescent	21	C580Y	108.92	Group 2

[1] For each patient, the clinical outcome was evaluated at day 42, after PCR-correction (distinction between reinfection and recrudescent in case of *P falciparum* recurrent infections) according to the 2009 WHO protocol.

[2] Mutation in the propeller domain of the k13 gene was assessed by nested PCR and sequencing (see ref 18).

[3] PSA survival rates (%) are expressed as the median of the survival rates (expressed as percent of viable parasites). Ex-vivo PSA assays were considered as interpretable if the growth rate in non-exposed culture (parasitemia at 72h/parasitemia at 0h) was ≥1. The limit of detection of viable parasites was estimated 0.1% parasitemia (number of red blood cells counted by each microscopist = 20,000 and average parasitemia in non-exposed = 5%).

[4] Classification of the day 0 isolates was done according to the following definition (see figure 3): Group 0 = patients harboring parasites with wild-type K13 allele and PSA ex-vivo survival rate < 10% at day 0: Group 1 = patients harboring parasites with mutant K13 allele and PSA ex-vivo survival rate < 10% at day 0 and Group 2 = patients harboring parasites with mutant K13 allele and PSA ex-vivo survival rate ≥ 10% at day 0. Patient # 6914 was unclassified.

[5] Day 0 isolate of the patient # 6914 was genotyped for K13 as wild-type 3D7 allele but the $RSA^{0\text{-}3h}$ survival rate, performed as previously (see appendix 1) on the same isolate was estimated to 16.9%, and considered as artemisinin resistant parasites (see ref 10).
