# Supplementary material for: Plasmodium falciparum dihydroartemisinin-piperaquine failures in Cambodia are associated with mutant K13 parasites presenting high survival rates in novel piperaquine in vitro assays: retrospective and prospective investigations
Source: BMC Med. 2015 Dec 22;13:305. doi: 10.1186/s12916-015-0539-5 (PMC4688949; doi:10.1186/s12916-015-0539-5)

Panel A. Isolates #6267 (on the left) and 3D7 (on the right. quintuple assays)

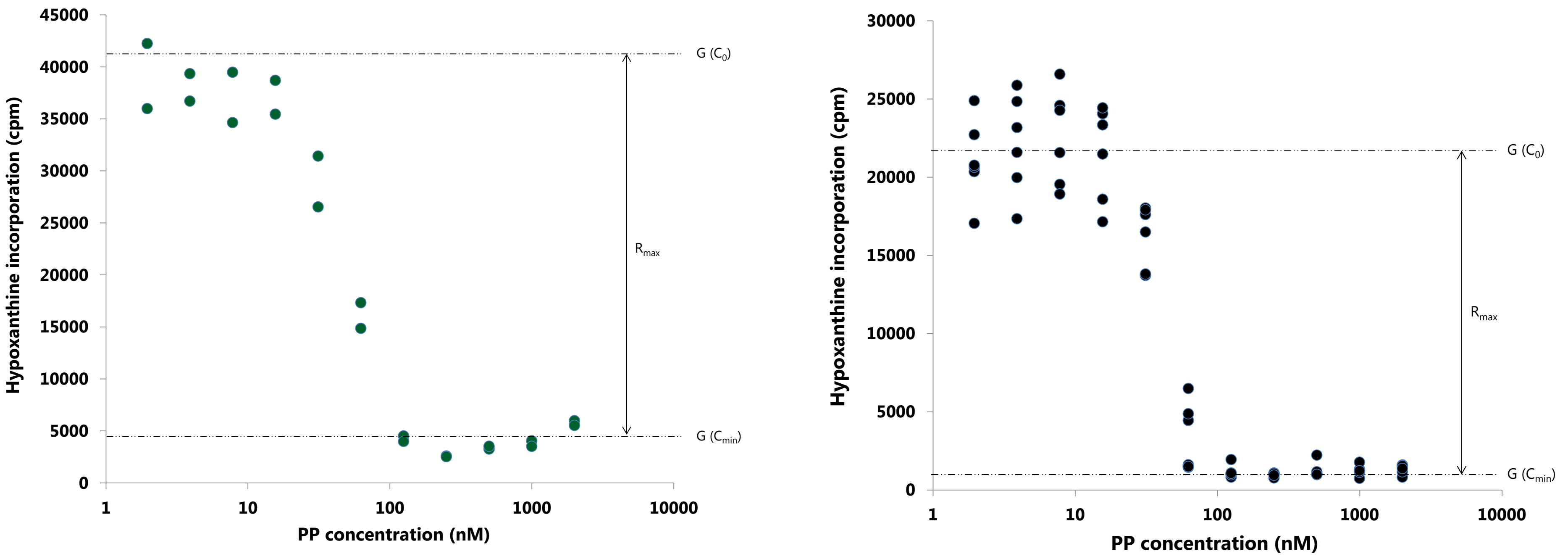

Panel B. Isolates #6272 (on the left) and # 6246 (on the right)

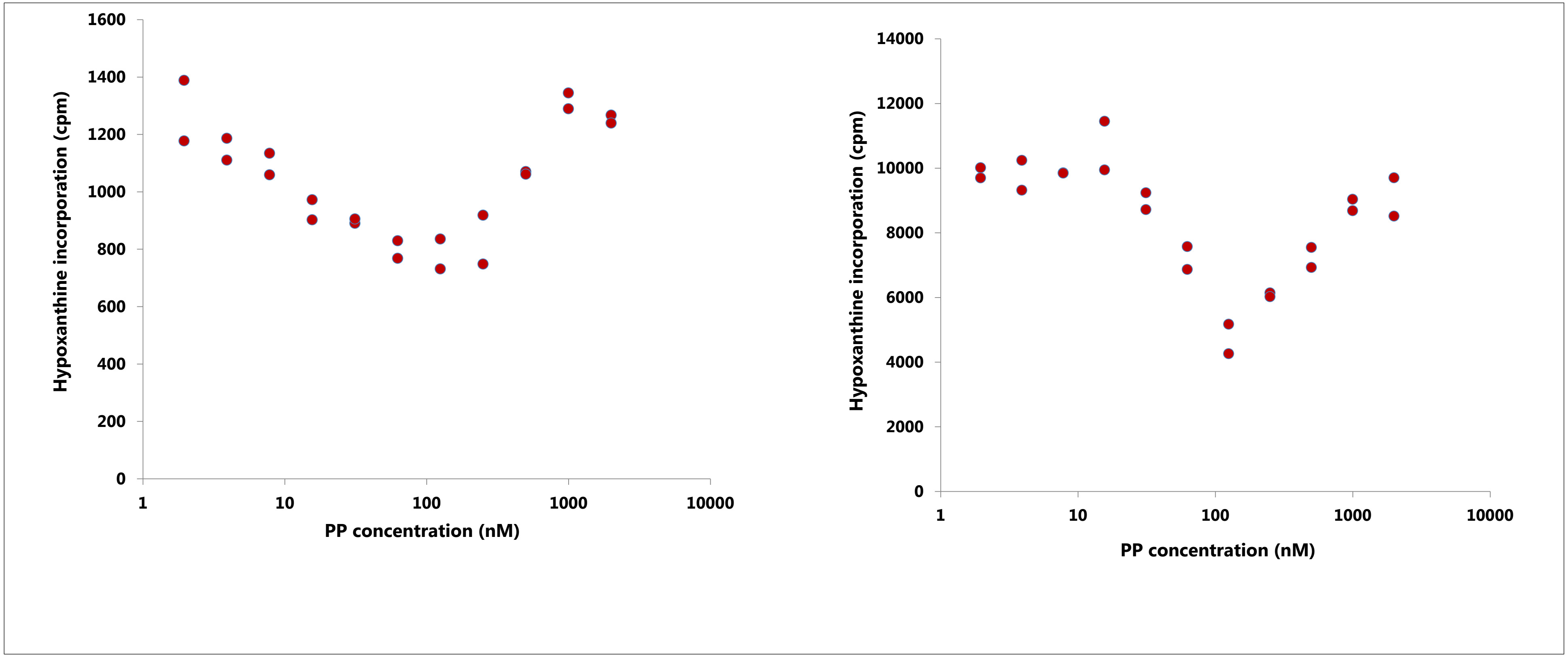

Panel C. Isolates #6218

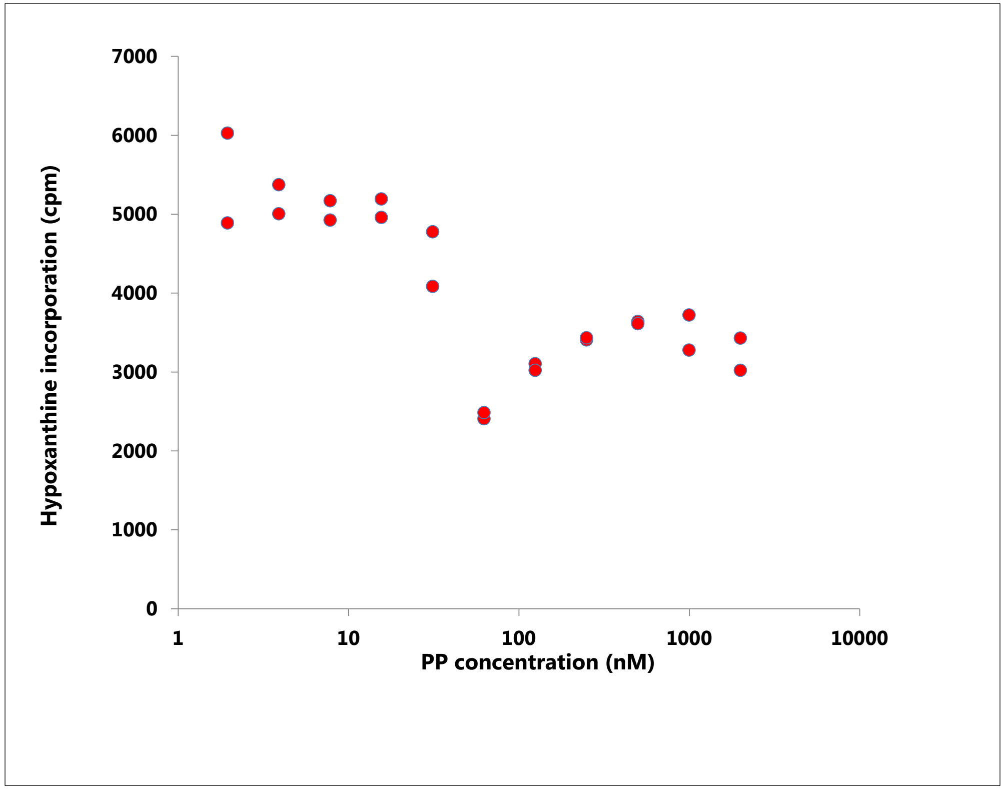

Supplement: Additional file 3: — Examples of interpretable piperaquine concentration-inhibition curves (Panel A) and non-interpretable piperaquine concentration-inhibition curves due to a paradoxical increase of apparent growth at high drug concentrations (Panel B: curves do not fit the data; Panel C: core criteria of curves are not acceptable). (PDF 40 kb) [file 12916_2015_539_MOESM3_ESM.pdf]
