# Supplementary figures and images for: Plasmodium falciparum dihydroartemisinin-piperaquine failures in Cambodia are associated with mutant K13 parasites presenting high survival rates in novel piperaquine in vitro assays: retrospective and prospective investigations
Source: BMC Med. 2015 Dec 22;13:305. doi: 10.1186/s12916-015-0539-5 (PMC4688949; doi:10.1186/s12916-015-0539-5)

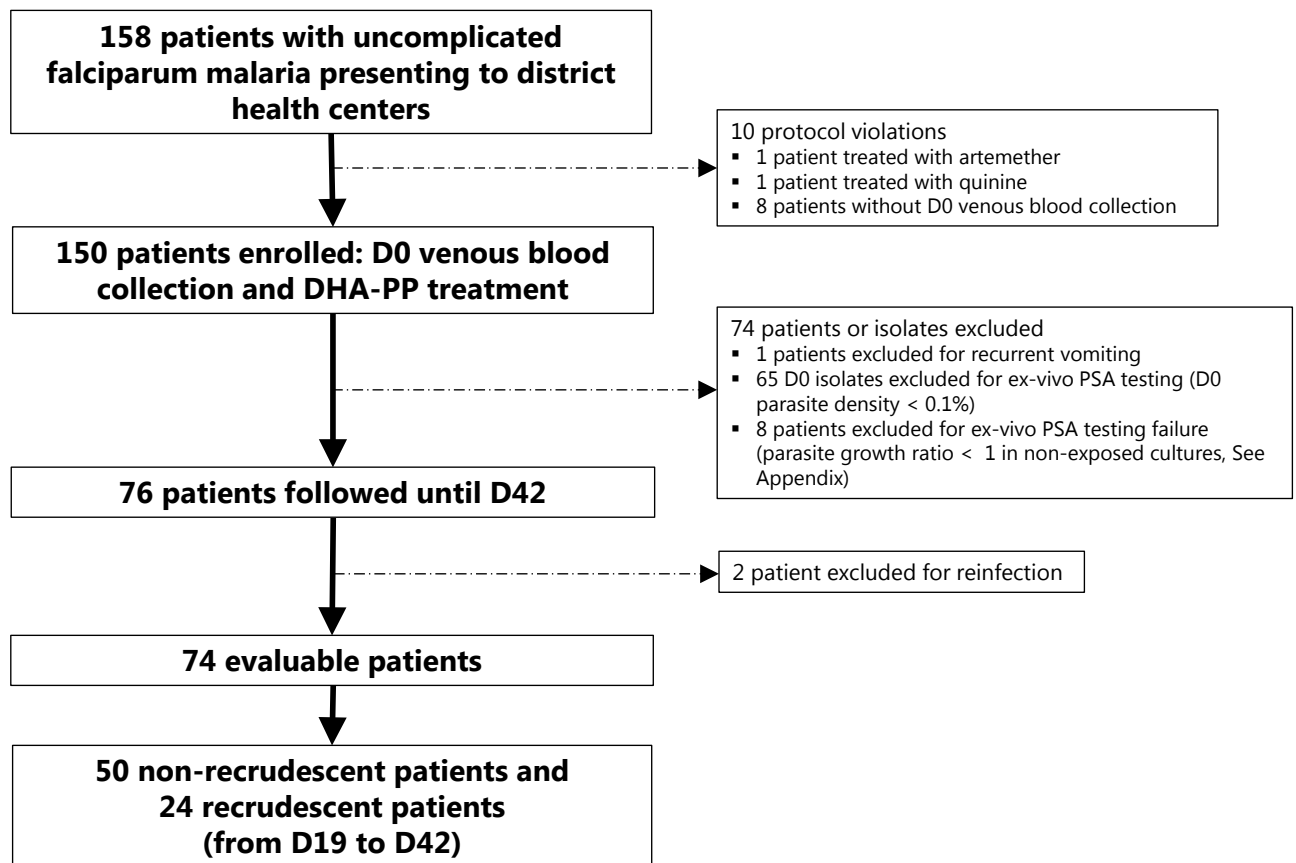

Supplement: Additional file 4: — Flow chart of patients included in the final analysis of the 2014 retrospective study. Cambodia 2014 and isolates used in ex vivo assays. (PDF 52 kb) [file 12916_2015_539_MOESM4_ESM.pdf]
