# Supplementary material for: Plasmodium falciparum dihydroartemisinin-piperaquine failures in Cambodia are associated with mutant K13 parasites presenting high survival rates in novel piperaquine in vitro assays: retrospective and prospective investigations
Source: BMC Med. 2015 Dec 22;13:305. doi: 10.1186/s12916-015-0539-5 (PMC4688949; doi:10.1186/s12916-015-0539-5)
Supplement: Additional file 5: — In vitro piperaquine survival assay survival rate distribution in 32 culture-adapted P. falciparum isolates from Cambodia in 2012 according to mutations or copy number variation in candidate genes significantly associated with piperaquine resistance (see Table 1 ). Panel A: mutations of P. falciparum chloroquine resistant transporter gene (Pfcrt); Panel B: copy number variation of P. falciparum multidrug resistance 1 gene (Pfmdr-1). (PDF 85 kb) [file 12916_2015_539_MOESM5_ESM.pdf]

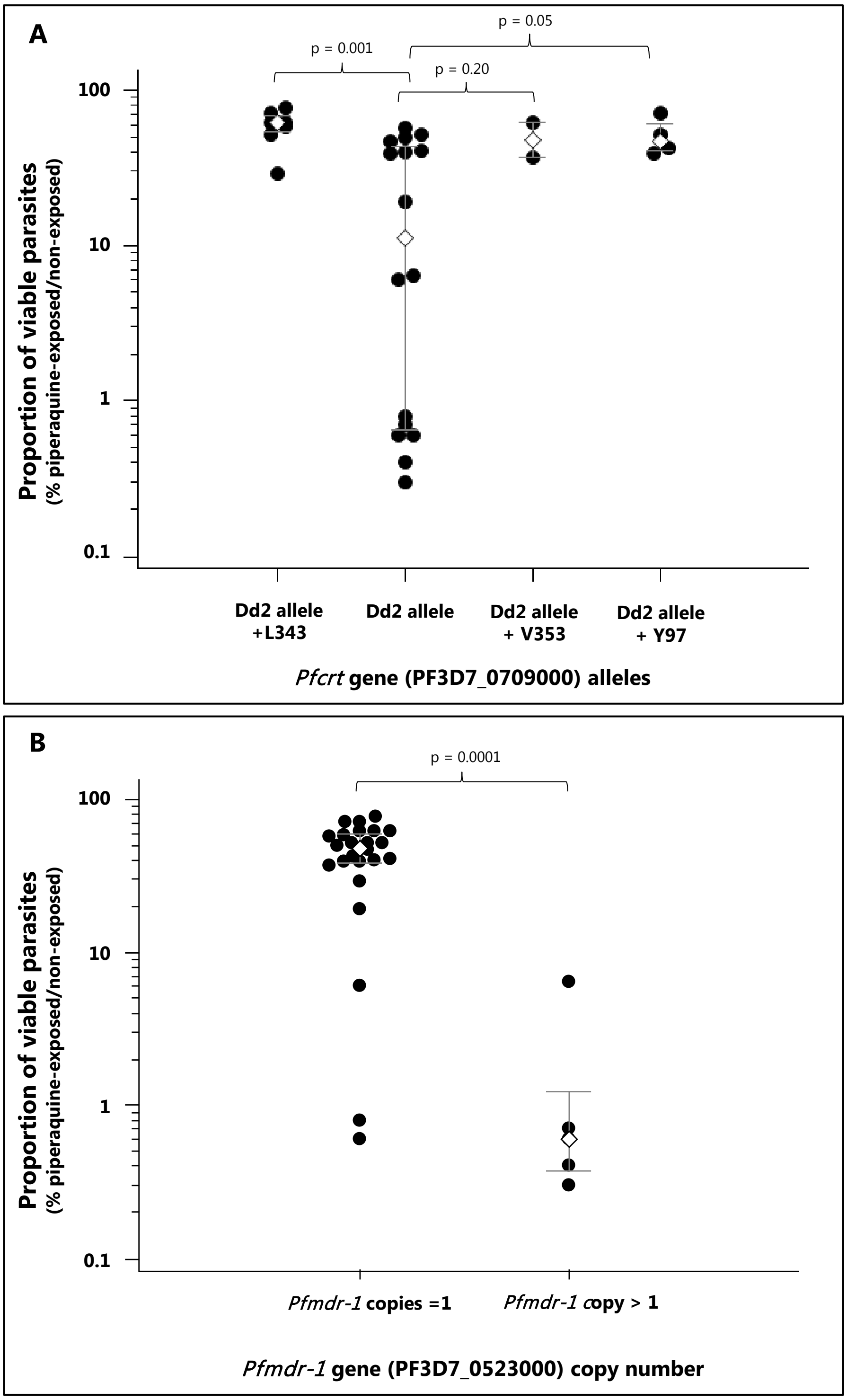

P-values for Mann-Whitney U test. The open diamonds, the horizontal lines and I bars represent the medians and interquartile ranges. All isolates with Pfmdr-1 > 1 copy are piperaquine-sensitive (in-vitro PSA survival rate <10%) but isolates with single-copy Pfmdr-1 are not all piperaquine-resistant (in-vitro PSA survival rate ≥10%).
