# Supplementary material for: Plasmodium falciparum dihydroartemisinin-piperaquine failures in Cambodia are associated with mutant K13 parasites presenting high survival rates in novel piperaquine in vitro assays: retrospective and prospective investigations
Source: BMC Med. 2015 Dec 22;13:305. doi: 10.1186/s12916-015-0539-5 (PMC4688949; doi:10.1186/s12916-015-0539-5)
Supplement: Additional file 6: — Results of the piperaquine concentration measurements in culture supernatants of different wells in plate used for the classical isotopic assay, containing increasing piperaquine concentrations (from 1.91 nM in PPQ11 to 2000 nM in PPQ1). (DOCX 12 kb) [file 12916_2015_539_MOESM6_ESM.docx]

| **ID** | **PPQ concentration (nM) used for the classical isotopic assay** | **PPQ detected in the culture supernatant (ng/ml)** |
| --- | --- | --- |
| PPQ1 | 2000 | 466 |
| PPQ2 | 1000 | 314 |
| PPQ3 | 500 | 167 |
| PPQ4 | 250 | 65.4 |
| PPQ5 | 125 | 23.2 |
| PPQ6 | 62.5 | 8.7 |
| PPQ7 | 31.25 | 3.86 |
| PPQ8 | 15.62 | 2.49 |
| PPQ9 | 7.81 | 1.6 |
| PPQ10 | 3.90 | <LOD |
| PPQ11 | 1.91 | <LOD |

*PPQ concentrations were determined from culture supernatant of different wells containing increasing PPQ concentration (from 1.91 nM in PPQ11 to 2000 nM in PPQ1). Solid-phase extraction was performed, followed by quantification by liquid chromatography and tandem mass spectrometry (multiple reaction monitoring [MRM] mode) detection on an AB Sciex API 5000 triple quadrupole mass spectrometer. Three controls were used (4.49 ng/ml, 20.3 ng/ml and 403 ng/ml). The limit of detection (LOD) was 0.375 ng/ml (signal-tonoise ratio,_3:1), and the lower limit of quantitation was 1.5 ng/ml for PPQ.*
